# Necrotic Thymoma Discovered Due to Subjective Symptoms: A Report of Three Cases

**DOI:** 10.70352/scrj.cr.25-0216

**Published:** 2025-06-18

**Authors:** Takuya Tokunaga, Naoko Ose, Hideki Nagata, Eiichi Morii, Yasushi Shintani

**Affiliations:** 1 Department of General Thoracic Surgery, Osaka University Graduate School of Medicine, Suita, Osaka, Japan; 2 Department of Pathology, Osaka University Graduate School of Medicine, Suita, Osaka, Japan

**Keywords:** necrotic thymoma, spontaneous regression, mediastinal tumor

## Abstract

**INTRODUCTION:**

Thymomas are solid tumors that usually grow slowly and rarely cause symptoms or spontaneously regression. We have observed three cases of thymoma in which the patient presented with fever and chest pain, and pathological examination showed relatively extensive necrosis. The tumors spontaneously shrank during the course of the diseases.

**CASE PRESENTATION:**

The patients, of a 30-year-old man, 46-year-old man, and 76-year-old man presented with fever and/or chest pain, and blood tests showed high levels of inflammation. Contrast-enhanced chest computed tomography (CT) showed masses with low-density area and contrast-enhanced margins. Two patients had repeat chest CT just prior to surgery, and the tumors had shrunk. In all cases, the masses were removed by a median sternotomy. The mediastinum tissue was hard due to inflammation, and in all cases the tumors were adherent to the lungs and in one case wedge resection of the left lung was required. Histopathological examination revealed extensive necrosis of the tumors, and based on residual viable tumor cells, the three tumors were diagnosed as follows respectively; type B2, type B2 with some type B3 components, and type AB thymoma. All tumors were classified as pT1aN0M0, Stage I, and Masaoka stage II.

**CONCLUSIONS:**

Necrotic thymoma is associated with inflammation and spontaneous regression may be observed during the course of the disease. Since necrosis can be extensive, pathological examination should be performed throughout.

## Abbreviations

CK
: cytokeratin
CRP
: C-reactive protein
CT
: computed tomography
CYFRA
: cytokeratin 19 fragment
FDG-PET
: ^18^F-fluorodeoxyglucose positron emission tomography
TdT
: terminal deoxynucleotidyl transferase

## INTRODUCTION

Thymomas are often found asymptomatically and rarely present with subjective symptoms.^1)^ There is a report that indicate thymoma is accompanied by changes such as necrosis, hemorrhage, and cystic degeneration,^2)^ but thymoma with extensive necrosis are uncommon.^3)^ On the other hand, it is usually a slowly enlarging, solid tumor that rarely shrinks spontaneously.^4)^ We experienced three cases of thymoma with relatively extensive necrosis, which were detected by chest pain and fever. In these cases, spontaneous regression of the tumor was also observed.

## CASE PRESENTATION

### Case 1

**Fig. 1:**
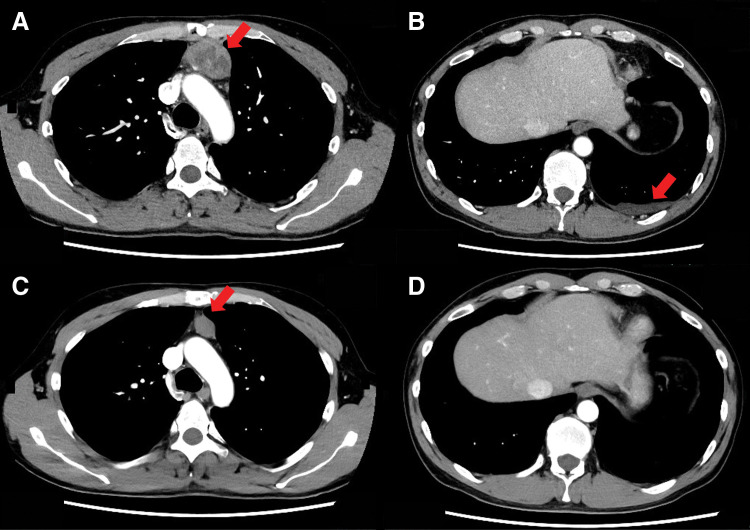
Case 1. Finding of chest computed tomography (CT). (**A**) Chest CT showed an 87 × 52 × 38 mm multilocular cystic tumor in the anterior mediastinum with contrast enhancement in the walls (arrow). (**B**) A small amount of pleural effusion was observed on the left side (arrow). (**C**, **D**) Chest CT 2 months after the first presentation revealed tumor regression (arrow) and disappearance of the pleural effusion.

**Fig. 2:**
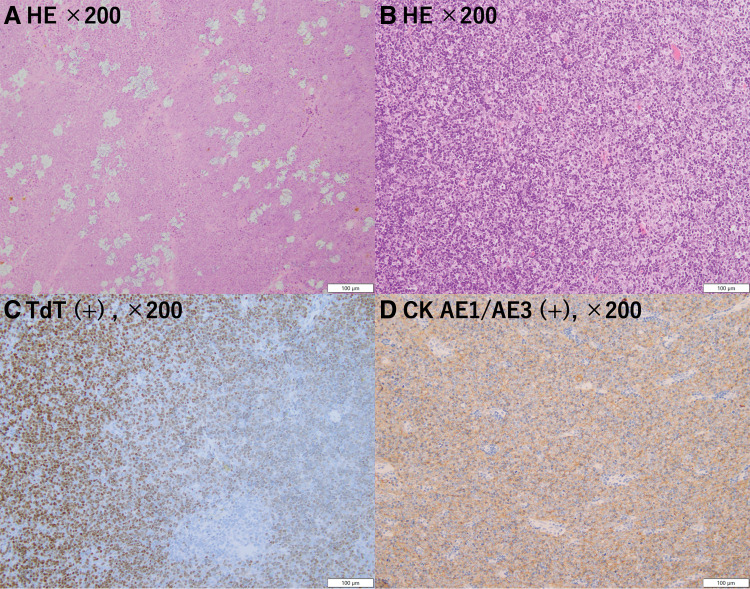
Case 1. Histological findings of the resected specimen. (**A**) Hematoxylin and eosin staining showed that the tumor was composed of an extended necrotic area. (**B**) Hematoxylin and eosin staining showed that the thymic epithelium was proliferating with lymphoid cells and tumor cells. (**C**, **D**) Immunohistochemical analyses with TdT and CK AE1/AE3 were positive in the tumor area.

A 46-year-old man presented with fever, sore throat, and chest pain and visited a local doctor. Blood tests showed a high C-reactive protein (CRP) level of 19 mg/dL, so he was prescribed oral antibiotics. Five days later, the symptoms tended to improve but persisted, and a CT scan revealed an anterior mediastinal tumor. He was referred to our hospital. Contrast-enhanced chest CT showed an 87 × 52 × 38 mm multilocular cystic mass in the anterior mediastinum with an inhomogeneous interior and contrast effect on the wall (**Fig. 1A**) and pleural effusion was observed predominantly on the left side (**Fig. 1B**). The CRP level improved, falling to 1 on the 14th day after onset, so the patient was observed until his overall condition stabilized. Two months after the onset of the symptoms, the chest CT showed that the mass had shrunk to 80 × 32 × 21 mm (**Fig. 1C**) and the left pleural effusion had disappeared (**Fig. 1D**). We considered teratoma and its rupture as a differential diagnosis. Resection of thymoma with partial thymectomy was performed by median sternotomy. The tumor was adhered to the left upper lobe, but could be detached. The operation time was 123 minutes, and blood loss was 30 mL. Pathological examination revealed that the tumor was extensively necrotic, with only a few areas containing viable tumor cells (**Fig. 2A**). The areas showed lymphocyte-dominated areas and areas of mixed proliferation of oval tumor epithelial cells and lymphocytes (**Fig. 2B**), and was positive for terminal deoxynucleotidyl transferase (TdT) (**Fig. 2C**) and cytokeratin (CK) AE1/AE3 (**Fig. 2D**). The patient was diagnosed as type B2 thymoma, pT1aN0M0, Stage I, and Masaoka stage II. The patient was discharged on the 6th postoperative day without any complications. Recurrence was not identified 13 months after surgery.

### Case 2

**Fig. 3:**
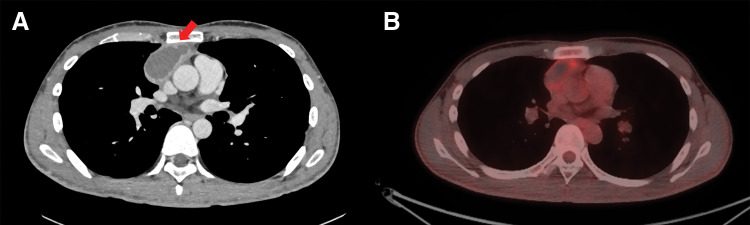
Case 2. Finding of chest CT. (**A**) Chest CT showed an 88 × 60 × 29 mm cystic tumor in the anterior mediastinum with contrast enhancement in the walls (arrow). Pleural effusion was not observed. (**B**) ^18^F-fluorodeoxyglucose positron emission tomography (FDG-PET) computed tomography shows FDG uptake with a maximum standardized uptake value of 3.2.

**Fig. 4:**
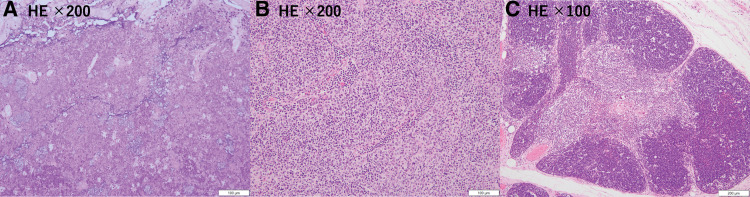
Case 2. (**A**) More than 75% of the tumor was necrotic. (**B**) Hematoxylin and eosin staining showed that the thymic epithelium was proliferating with lymphoid cells and tumor cells. (**C**) Hematoxylin and eosin staining showed that germinal center formation was observed in the thymus and it was hyperplastic.

A 30-year-old man had a fever of 39 degrees, and 2 days later he had right anterior chest pain and exertional dyspnea. Blood tests showed elevations of cytokeratin 19 fragment (CYFRA) (4.2 U/mL), CRP (1.84 mg/dL), and anti-acetylcholine receptor antibody (3.2 pmol/mL). The patient exhibited no myasthenic symptoms. Contrast-enhanced chest CT showed an 88 × 60 × 29 mm cystic mass in the anterior mediastinum with irregularly contrasted margins (**Fig. 3A**) and shadows suggestive of pneumonia in the right upper lobe and bilateral lower lobes. ^18^F-fluorodeoxyglucose positron emission tomography (FDG-PET) revealed FDG uptake with a maximum standardized uptake value of 3.2. (**Fig. 3B**). Two months after the onset of symptoms, extended thymectomy was performed by a median sternotomy. The tumor was adherent to the right upper and middle lobe, but could be detached. The mediastinum tissue was hard due to inflammation. The operation time was 125 minutes, and blood loss was 75 mL. Pathological examination revealed most of the tumor was necrotic, indicating degeneration of tumor cells (**Fig. 4A**). Outside the necrotic area, proliferation of lymphocytes and oval tumor cells were seen (**Fig. 4B**), which was diagnosed type B2 thymoma. There was a type B3 component, and capsular infiltration was observed. The thymic tissue was hyperplastic with a germinal center formation (**Fig. 4C**). The tumor classified as pT1aN0M0, Stage I and Masaoka stage II. Postoperatively, pneumonia was observed in the right lower lobe, but blood tests showed normal IgG levels. The patient remains recurrence-free with no symptoms of myasthenia gravis at 11 months after surgery.

### Case 3

**Fig. 5:**
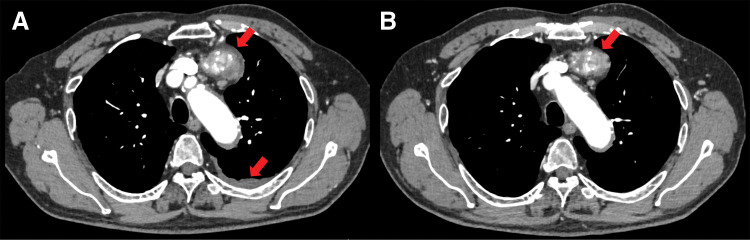
Case 3. Finding of chest CT. (**A**) Chest CT showed a 74 × 66 × 58 mm tumor with internal low-density area and contrast enhancement at the margins in the anterior mediastinum (arrows). A small amount of pleural effusion was observed on the left side. (**B**) Chest CT about 2 months after the first presentation revealed tumor regression (arrow) and disappearance of the pleural effusion.

**Fig. 6:**
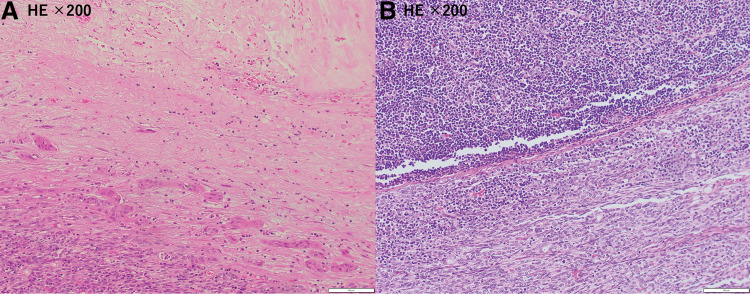
Case 3. (**A**) More than 75% of the tumor was necrotic. (**B**) Hematoxylin and eosin staining showed areas of lymphocyte-based formation and areas of spindle-shaped epithelial cells.

A 76-year-old man presented with dyspnea and chest pain. Blood tests showed elevated white blood cell counts of 10810/μL and CRP of 19.7 mg/dL. Contrast-enhanced chest CT showed a 74 × 66 × 58 mm anterior mediastinal tumor with calcification, internal low-density area, and contrast effect at the margins (**Fig. 5A**) and pleural effusion was observed predominantly on the left side. He was admitted to the hospital immediately. After 8 days of intravenous antibiotic treatment, both his symptoms and the inflammatory findings in his blood tests improved, so he was discharged. About 2 months later, the CT showed that the mass had shrunk slightly to 68 × 59 × 51 mm (**Fig. 5B**), and the left pleural effusion had disappeared. Necrotic thymoma was also considered, in addition to teratoma. Resection of thymoma with partial thymectomy with median sternotomy and wedge resection of the left upper lobe was required because the tumor was attached to the left upper lobe firmly. The operation time was 250 minutes, and blood loss was 140 mL. Pathological examination revealed that most of the tumor was necrotic (**Fig. 6A**), and areas of lymphocyte-based formation and areas of spindle-shaped epithelial cells were seen (**Fig. 6B**). The tumor was classified as thymoma type AB, pT1aN0M0, Stage I, and Masaoka stage II. The postoperative course was uneventful, and the patient was discharged on day 12. There was no apparent recurrence during a follow-up visit after 2 months.

## DISCUSSION

Thymoma with extensive necrosis is also called necrotic thymoma.^5)^ Although there is no clear definition of necrotic thymoma, imaging findings in our cases showed that at least half of the tumor was composed of necrotic tissue. Necrotic thymoma is rare and it has been reported in only 20 cases.^3–18)^ The 23 cases including the present cases are shown in **Table 1**; 11 cases had fever and 21 cases had chest pain. Chest CT images showed pleural effusions in 16 cases. Most patients were classified as having early-stage (stage I: 7, stage II: 12, stage III: 1, stage IV: 1), and there were few reports of recurrence. The cause of the chest pain is thought to be inflammation due to tumor necrosis that spread to pleurisy, which is supported by the presence of pleural effusion, hard changes in the mediastinal tissue, and adhesions to the lungs. Symptom onset was the trigger for thymoma detection, and even small tumors are considered to be accompanied by necrosis.

**Table 1:** Previous case reports of thymoma with extensive necrosis

Author	Year	Sex	Age	Chief complaint		WBC	CRP (mg/mL)	Size (mm)	SR	Pleural effusion	Histopathology	WHO type	Masaoka’s stage	Recurrence (months)
				Fever	Chest pain						Severe necrosis			
Wright and Wain^6)^	2006	F	51	○	○	–^§^	–	–	–	○	○ (≤95%)	B1	II	NED (69)
		M	39	–	○	–	–	–	–	○	○ (≥95%)	B1	II	NED (47)
		M	28	–	○	–	–	–	–	○	○ (>90%)	B2 or B3	II	NED (24)
Itoh et al.^7)^	2006	M	25	○	○	11500	8.4	100	○	○	○ (Tumor = 5%)	B1	I	–
Okagawa et al.^8)^	2007	F	31	–	○	13520	4.2	50	○	–	○	B2	II	–
Hori et al.^9)^	2008	M	38	–	○	7920	1.74	35	○	○	○	B2	II	NED (9)
Yutaka et al.^10)^	2008	M	47	–	○	W.N.L.	W.N.L.	80	○	○	○	B3	III	NED (12)
Agackiran et al.^11)^	2010	F	38	–	○	W.N.L.	–	90	–	–	○	A	–	NED (12)
Fukui et al.^12)^	2014	F	43	–	△^††^	–	–	34	○	○	○	B2	II	–
		F	32	○	○	–	–	100	○	○	○	B2	IV	–
Palma et al.^5)^	2014	F	58	–	○	–	–	–	×	○	○	–	–	NED (24)
Furuya et al.^13)^	2015	M	30	○	○	11000	4.1	110	○	○	○	B2	II	NED (12)
Kataoka et al.^3)^	2015	F	52	○	○	W.N.L.	6.54	55	–	○	○ (≥95%)	B1	I	NED (24)
Kasai and Masuya^14)^	2016	M	28	○	○	11400	4.93	35	○	–	○	B2 + B3	II	NED (63)
Yasukawa et al.^4)^	2017	F	71	○	○	W.N.L.	W.N.L.	100	○	○	○	A	I	NED (16)
		F	59	–	–	W.N.L.	W.N.L.	60	○	–	○	B2	I	NED (17)
Kim et al.^15)^	2018	M	43	–	○	–	–	45	○	–	○	Unknown^§§^	I	NED (6)
Sakaguchi et al.^16)^	2019	M	38	○	○	11200	3.38	100	○	○	○	B1	I	24 after surgery
Hinokuma et al.^17)^	2020	M	36	○	○	14830	1.64	105	●^‡^	○	○	B2	II	NED (12)
Yasuda et al.^18)^	2021	M	49	–	○	–	–	60	–	×	○	B2	II	–
Our cases	2025	M	46	○	○	W.N.L.	14.54	87	○	○	○	B2	I	NED (13)
		M	30	○	○	W.N.L.	1.84	88	●	×	○	B2 + B3	II	NED (11)
		M	76	×	○	10810	19.7	74	○	○	○	AB	II	NED (2)

–^§^, not applicable; △^††^, right shoulder pain and back pain; ●^‡^, compare CT to specimen; unknown^§§^, not identified due to severe necrosis.

WBC, white blood cell; CRP, C-reactive protein; SR, spontaneous regression; WHO, World Health Organization; NED, no evidence of disease; W.N.L., within normal limits

Necrotic thymoma is sometimes difficult to diagnose because the tumor area is small. There is a report of a case in which a mass could not be diagnosed by CT-guided biopsy before surgery. Even if the diagnosis cannot be confirmed by biopsy, it is appropriate to choose surgical treatment with the possibility of thymoma in mind when extensive necrosis is suspected.^17)^ In Case 1, there were few viable tumor cells, and the diagnosis was made finally by microscopic observation of the entire tumor. Therefore, it should be noted that anterior mediastinal tumors with extensive necrosis require surgical resection followed by pathological examination of the entire specimen.

On the other hand, there are relatively few reports of thymomas that shrink spontaneously.^4)^ Yasukawa et al.^4)^ reported 21 cases of thymoma with spontaneous regression and its characteristics were similar to necrotic thymoma such as symptoms like chest pain and fever, relatively early stage, and necrosis and fibrosis on histopathology. A total of 15 of the 23 cases in **Table 1** showed spontaneous regression.^4,7–10,12,14–17)^ Okagawa et al.^8)^ also found a relationship between necrosis and spontaneous regression, and speculated that necrosis is caused by bloodstream disorder due to rapid tumor growth for some reason. On the other hand, even in cases in which rapid tumor growth is not observed, the tumor itself is accompanied by inflammation with lymphocyte infiltration, which can easily cause thrombosis and lead to necrosis.^9)^ In our cases, we did not observe enlargement of the tumors, but clusters of lymphocytes were found in areas lacking tumor epithelial cells around the necrotic tissue in all cases. Spontaneous regression is reported to occur when necrosis and hemorrhage are absorbed.^10)^ It is therefore assumed that necrosis and spontaneous regression in thymoma are closely related, and that they may be on the same spectrum.

It is also reported in cases of teratoma with perforation to show severe inflammatory findings and to shrink during the course of the disease.^19)^ In Case 3, the tumor is accompanied by calcification, which is an imaging feature seen in teratomas as well. It is difficult to distinguish between necrotic thymoma and teratoma with perforation based on clinical findings, thus surgical treatment is necessary for diagnosis for patients with anterior mediastinal tumors accompanied by fever and chest pain.

## CONCLUSIONS

Three cases of thymoma with subjective symptoms revealed extensive necrosis. Necrotic thymoma is associated with inflammation and spontaneous regression may be observed during the course of the disease. Diagnostic surgical resection may be useful, and since necrosis can be extensive, pathological examination should be performed throughout.

## DECLARATIONS

### Funding

This study was not funded.

### Authors’ contributions

TT and NO described and designed the article.

NO is the corresponding author.

YS supervised the edition of the manuscript.

EM and HN performed the histopathological diagnosis.

All collected the data and discussed the content of the manuscript and read and approved the final manuscript.

### Availability of data and materials

All data generated or analyzed during this study are included in this published article.

### Ethics approval and consent to participate

The study protocol was approved by the Ethical Review Board for Clinical Studies of Osaka University (Control No.: 10026-3).

### Consent for publication

Informed consent for publication of this case report was obtained from the patient.

### Competing interests

The authors declare that they have no competing interests.
